# Design of an harmonic radar for the tracking of the Asian yellow‐legged hornet

**DOI:** 10.1002/ece3.2011

**Published:** 2016-03-02

**Authors:** Daniele Milanesio, Maurice Saccani, Riccardo Maggiora, Daniela Laurino, Marco Porporato

**Affiliations:** ^1^ Dipartimento di Elettronica e Telecomunicazioni (DET)Politecnico di TorinoTorinoItaly; ^2^ Dipartimento di Scienze Agrarie, Forestali e Alimentari (DISAFA)Universitá degli Studi di TorinoTorinoItaly

**Keywords:** Asian hornet, harmonic radar, insect tracking, *Vespa velutina*

## Abstract

The yellow‐legged Asian hornet is an invasive species of wasps, indigenous to the Southeast Asia but recently spreading in Southern Europe. Because of its exponential diffusion and its serious threat to the local honeybee colonies (and to humans as well), restraint measures are currently under investigation. We developed and tested an harmonic radar capable of tracking the flying trajectory of these insects, once equipped with a small transponder, in their natural environment. Several hornets were captured close to a small cluster of honeybee hives, tagged with different transponders and then released in order to follow the flight toward their nest. On‐field testing proved an initial maximum detection range of about 125 m in a hilly and woody area. A number of detections were clearly recorded, and preferential directions of flight were identified. The system herein described is intended as a first low‐cost harmonic radar; it proved the capability to track the hornets while flying and it permitted to test the tagging techniques. Several upgrades of the system have been identified during this work and are extensively described in the last chapter. The designed system has three major advantages over conventional harmonic radars. First and most importantly, it adopts advanced processing techniques to suppress clutter and to improve target detection. Second, it allows radar operations in complex environments, generally hilly and rich in vegetation. Finally, it can continuously track tagged insects (24/7) and in any meteorological condition, providing an effective tool in order to locate the nests of the yellow‐legged Asian hornet.

## Introduction

**Figure 1:**
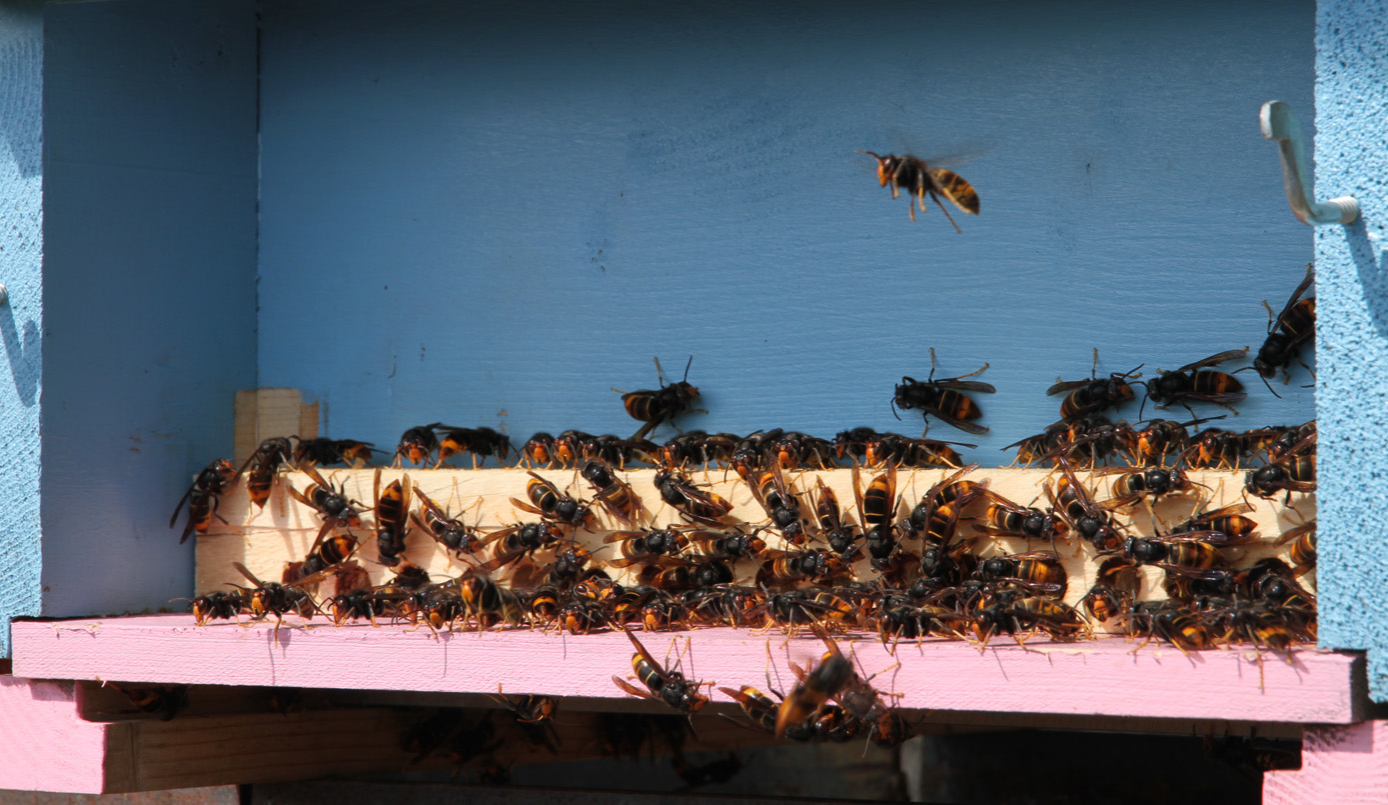
A honeybee hive in Dolceacqua (Italy) assaulted by a group of yellow‐legged hornets during a sunny afternoon of summer 2015. The bees present inside the hive, if any alive, cannot get out safely.

The yellow‐legged Asian hornet, also known as *Vespa velutina* Lapeletier, 1836, is a kind of predatory hornet originally from the Southeast Asia; it arrived in France probably in 2004 (Haxaire et al. 2006) and is currently quickly spreading in Southern Europe (Rome et al. 2013; Demichelis et al. 2014). This giant wasp is not only extremely dangerous for the humans, but it can be considered a lethal threat to honeybees, whose colonies, being a perfect source of proteins for its larvae, are decimated during summer time; Figure 1 shows a honeybee hive besieged by a numerous group of yellow‐legged hornets on a sunny day in July 2015 in Dolceacqua (Italy). Due to its quick growth rate (every queen generates a maximum of 563 queens in a year, as outlined in Rome et al. 2015), standard trapping methods proved to be not quite effective to limit its diffusion; conversely, locating its nests, and then destroying them, may be considered a viable approach to its containment. However, being the *V. velutina* nests usually built on trees, their localization is definitely not trivial, especially on hilly and woody areas such as the inland of the Ligurian coast, the Italian region where the spreading is currently mainly taking place.

To successfully tackle this problem, we developed an harmonic radar system which is able to follow the flight of properly tagged specimens of the yellow‐legged Asian hornet. Harmonic radars have been used to track various insects for almost 30 years now, starting from the pioneering work reported in Mascanzoni and Wallin (1986), up to the more recent application described in Tsai et al. (2013). In most of the cases, its usage was motivated by the entomological interest in better knowing the habits of the observed insect (for instance in Osborne et al. 1999), but in some applications, like ours, prevailed the need of protecting the environment from invasive species (for instance in Psychoudakis et al. 2008).

## Materials and Methods

**Figure 2:**
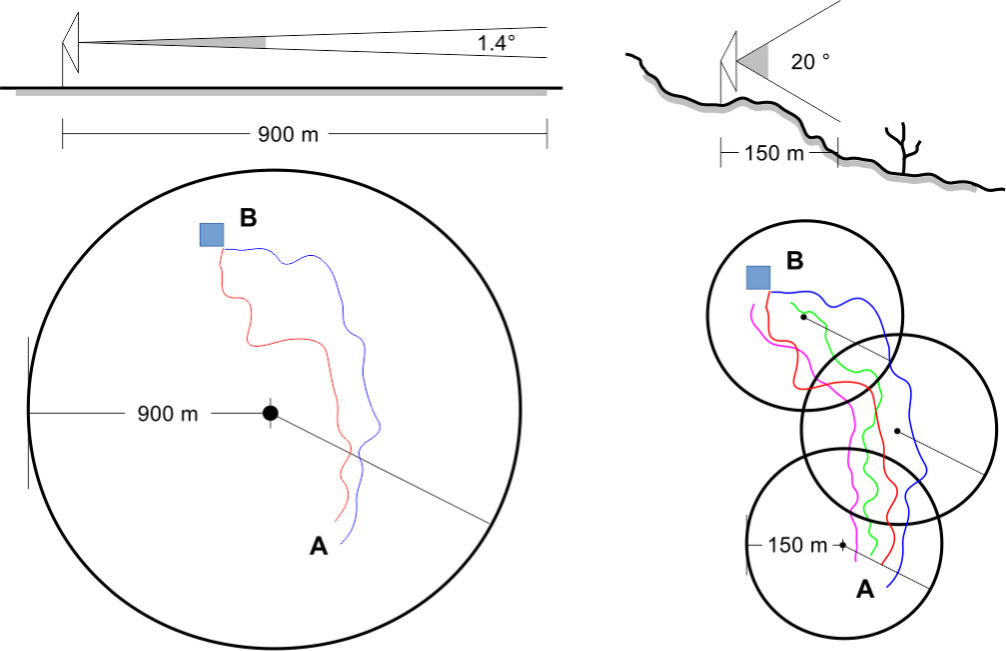
Example of a typical radar acquisition as described in Riley and Smith (2002) (left) and in this paper (right). In Riley and Smith (2002), the favorable flat environment allows to reduce the beam width of the TX and RX antennas and, therefore, to increase the detection range; the radar is located in between the release point A and the hive position B. The work herein presented requires a broader beam width in order to operate in a hilly and woody environment, with a consequent reduction of the detection range; multiple detections are then needed to find the Asian hornet nest in B starting from the release point in A.

Like all harmonic radars, no matter the application field, also this system launches waves at a specific frequency and receives one of the harmonics (usually the stronger, i.e., the second) produced by a nonlinear device (usually a diode), which is mounted on the so‐called radar targets. That said, the operative environment in which our radar system is supposed to be working is much more challenging than most of the known entomological applications, for instance the one described in Riley and Smith (2002). In that specific work, the radar was operated in a flat scenario, absent from any obstacle that could prevent the screening of the transponder applied on the back of the insects. This favorable condition allowed the usage of highly directive antennas, namely two parabolic reflectors with a circular beam of 1.4${}^{\circ }$ half‐power beamwidth and 41.6 dBi gain; as a consequence, the range of that system was declared rather impressive, about 900 m. Figure 2 reports on the left a schematic visualization of the operative conditions in Riley and Smith (2002), where point A represents the release location of the insects and point B is the hive position.

With respect to the aforementioned experiment, the harmonic radar herein presented has the aim of following the flight of the yellow‐legged hornet up to its nest, in an environment which is usually hilly and rich in trees and tall bushes. Therefore, we had to provide more coverage in elevation, adopting antennas with a larger beam on the vertical plane and, consequently, a smaller range of operations. Figure 2 shows on the right the typical working conditions in which this radar system has been tested and optimized; this time, point A usually corresponds to a honeybee hive (where the hornets are captured) and B represents the position of the hornets' nest.

As the reader can notice, our system is based on a statistical analysis of several radar traces, basically because the presence of multiple obstructions will sometimes screen the signal coming from the tag mounted on the insects. However, with a statistically relevant number of tracked flights, we expect to be able to identify a preferential direction of flight which should correspond to the position of the nest. As the hornets do not fly too far from their nest, by moving the radar system and repeating the acquisitions along the preferential direction, we expect to find the nest location within few iterations.

**Figure 3:**
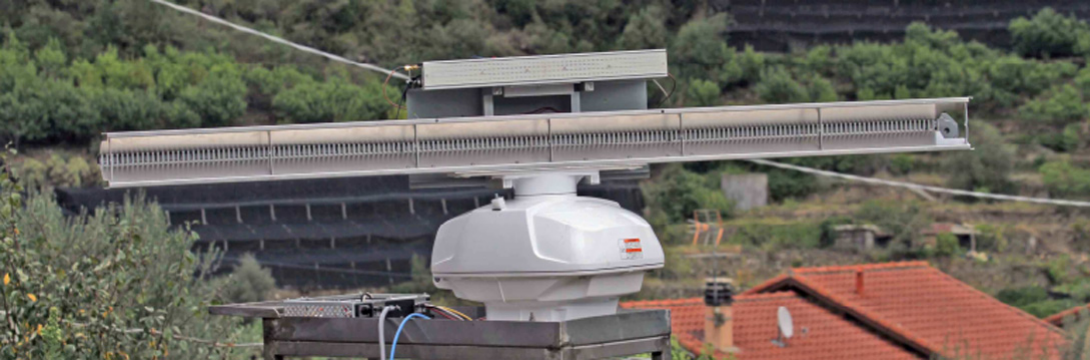
Harmonic radar for the yellow‐legged hornet tracking. The TX antenna is 180 cm long, while the RX antenna (on top of it) is 50 cm long.

To conclude this introductory part, Figure 3 reports the harmonic radar developed for this purpose. A 12‐V car battery, adopted to power all electronics, and a laptop, required to perform the analysis of the received signals, complete the system.

### Description of the TX system

**Figure 4:**
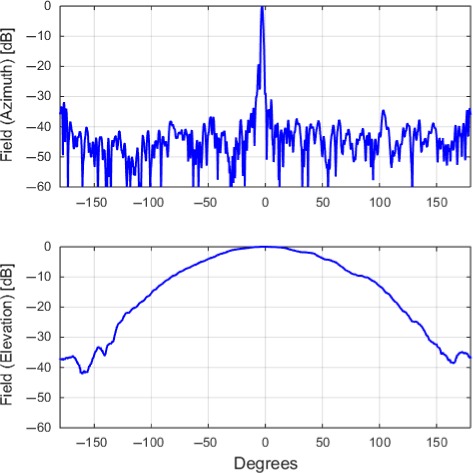
Radiated fields in azimuth (top) and elevation (bottom) of the TX antenna.

To keep the cost of the system at a reasonable level, the transmission module has been derived from a commercial off‐the‐shelf marine radar built by FURUNO, specifically the DRS25A model (http://www.furuno.com). It is essentially a 25‐kW magnetron pulsed generator transmitting a fixed frequency of 9.41 GHz, equipped with a horizontally polarized mechanically rotating slotted waveguide antenna 180 cm long; Table 1 lists the relevant features of the TX system configuration, while Figure 4 documents the radiation diagram of the TX antenna. The generator has been configured to operate with the minimum pulse width to maximize the range resolution and with the higher pulse repetition frequency to improve the detectability. The rotations per minute are fixed to 48.

**Table 1:** Features of DRS25A FURUNO marine radar adopted as the transmitting module of the system

Peak output power	25 kW
Pulse width	100 ns
Pulse repetition frequency (PRF)	3 kHz
Rotations per minute (RPM)	48
Frequency	9.41 GHz
Antenna hor. half‐power beam width (HPBW)	1.4${}^{\circ }$
Antenna vert. half‐power beam width	22${}^{\circ }$
Antenna gain	28.5 dBi
Antenna polarization	Horizontal

### Description of the RX system

**Figure 5:**
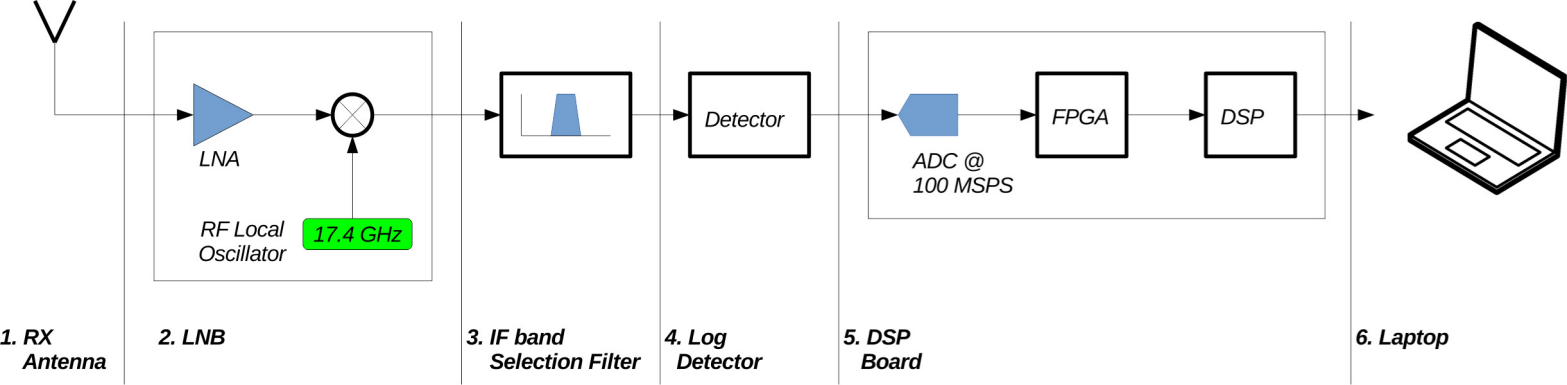
Schematic representation of the receiving chain. The signal received by the antenna is first amplified and then mixed with the local oscillator of a low‐noise down‐converter block (LNB). The demodulated signal is afterward filtered in the working band of the radar and then rectified by a logarithmic detector. The so‐obtained rectified signal is then digitized, buffered, and processed by a high‐performance digital signal processor (DSP) board. To conclude, the output data are eventually transferred to a laptop for the real‐time graphical visualization.

While the transmission part of the system was derived from a commercially available marine radar, the receiving module had to be assembled with ad hoc designed elements and commercially available components. Figure 5 schematically depicts the receiving chain, starting from the RX antenna on the left up to the laptop on the right. While herein providing a general description of the system, we invite the interested reader to the correspondent paragraphs for further details. For sake of simplicity also the transponder description is assumed to be part of the RX system.

The 18.82 GHz (2,× 9.41 GHz) signal received by the antenna is first amplified and then mixed with the local oscillator of a low‐noise down‐converter block (LNB) commercially adopted for satellite applications. The demodulated signal is afterward filtered in the working band of the radar and then rectified by a logarithmic detector. The so‐obtained rectified signal is then digitized, buffered, and processed by a high‐performance digital signal processor (DSP) board. To conclude, the output data are eventually transferred to a laptop for the real‐time graphical visualization.

#### The transponder

The adopted transponder is made up of two copper pieces connected to a zero bias Schottky diode that generates harmonics of the received signal, that is, from 9.41 to 18.82 GHz, and retransmit it back to the receiving station.

**Figure 6:**
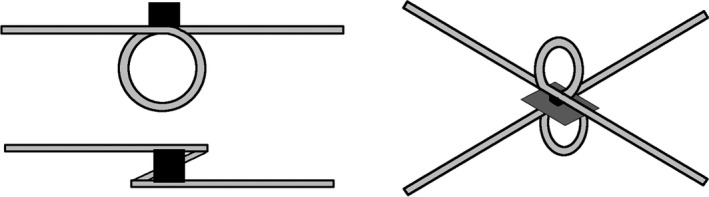
Schematic drawing of the tested tags: “loop" (left) and “cross" (right) configurations are shown. In case of the left configuration, when the tag polarization is orthogonal to the radar antennas polarization, there is no signal at all impinging on the transponder and, therefore, no retransmitted signal to the base station. This limitation is fully removed with the right configuration, that is, by mounting together two “loop" tags in orthogonal relative position.

The diode is manufactured by Skyworks with part number SMS7630‐079LF and wrapped in a rigid plastic package (http://www.skyworksinc.com/Product/511/SMS7630_Series#;). Starting from the work described in Colpitts and Boiteau (2004), we investigated several geometries and we eventually identified a better performing solution: Figure 6 on the left shows the "loop" geometry. Keeping constant the “loop" configuration, we also varied the main construction parameters, namely the diameter and the length of the copper wire and the central loop diameter. The best configuration was found with a 0.25‐mm‐diameter wire (in general, the thinner the wire, the lighter the transponder) and with a total length of 16 mm, with symmetrical branches on both sides of the diode. The loop across the diode is necessary because the rectifying action creates an electrostatic charge distribution between the two halves of the transponder of sufficient magnitude to bias the diode into a nonconducting state; for this element, the optimum is obtained with a 2.5 mm diameter. Finally, the global weight of the transponder is 12 mg.

We first estimated the conversion efficiency of the transponder using a signal generator as transmitter, two calibrated horn antennas, a spectrum analyzer as receiver and the transponder placed at 0.6 m of distance in front of the antennas. We used the harmonic cross section $\sigma_{h}$ defined in Colpitts and Boiteau (2004) and expressed as:

(1) $$\sigma_{h}=A_{\mathrm{df}}E_{d}G_{\mathrm{dh}}=\frac{\lambda_{f}^{2}}{4\pi }G_{\mathrm{df}}E_{d}G_{\mathrm{dh}}$$

where $A_{\mathrm{df}}$ is the effective area of the tag dipole at the fundamental frequency, $E_{d}$ is the conversion efficiency, $G_{\mathrm{dh}}$ is the gain of the tag dipole at the harmonic frequency, $\lambda_{f}$ is the wavelength at the fundamental frequency, and $G_{\mathrm{df}}$ is the gain of the tag dipole at the fundamental frequency. At the received power density of 200 mW/m${}^{2}$, the transponder shows an harmonic cross section $\sigma_{h}$ of about 2 mm${}^{2}$ with a conversion efficiency $E_{d}$ of the diode of about 0.65%, assuming $G_{\mathrm{dh}}$ equal to 2.41 and $G_{\mathrm{df}}$ equal to 1.64. The harmonic radar cross‐section pattern was measured for different positions of the transponder with respect to the TX and RX antennas and, as a first approximation, we verified that its behavior is quite similar to an electric half‐wavelength dipole.

**Figure 7:**
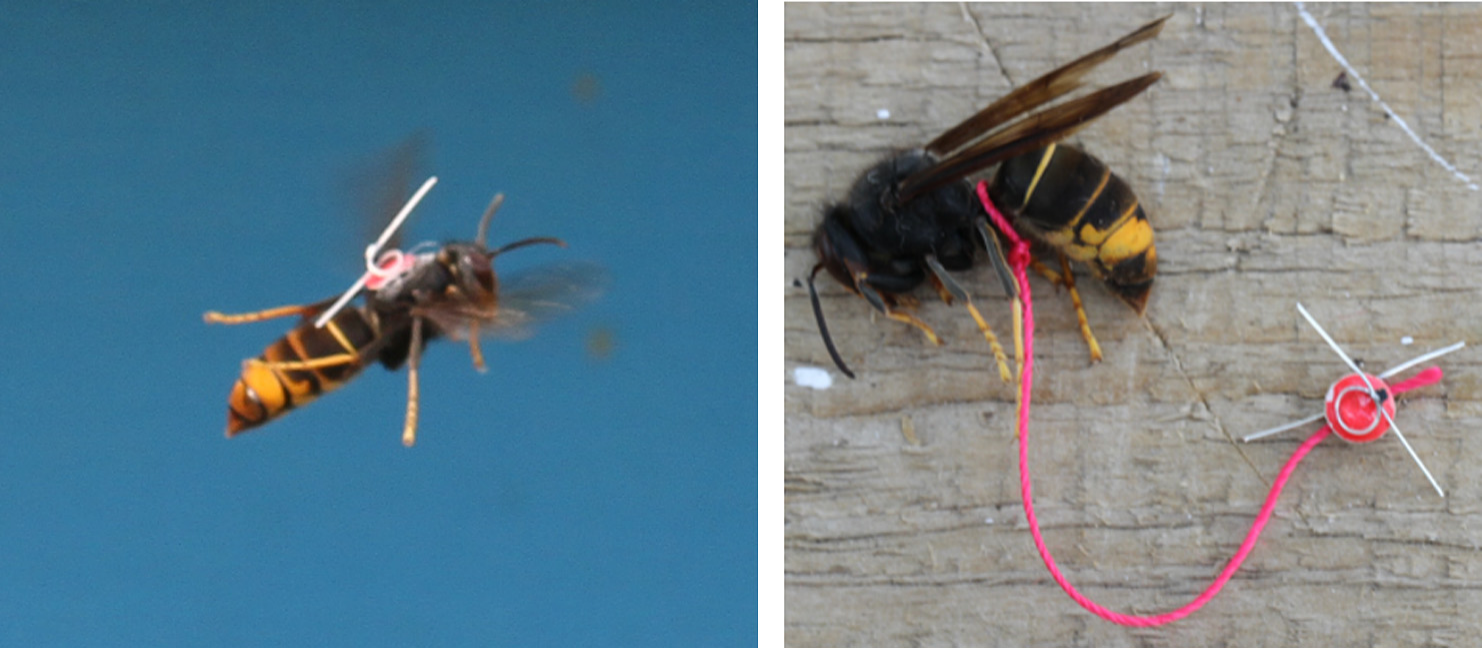
Yellow‐legged hornet equipped with a “loop" tag on its back (left) and with a “cross" tag hanging from the body (right). The length of the tag is 16 mm.

One final comment should be done before introducing the second tag configuration, namely the “cross" geometry (see Fig. 6 on the right). As both the transmitting and the receiving antennas work in horizontal polarization (see next sections for further details), the adoption of a single tag limits the tracking, above all if the transponder is glued to the back of the insect, as documented in Figure 7 on the left; as a matter of fact, when the tag polarization is orthogonal to the radar antennas polarization, that is, when the hornet is flying perfectly to or from the radar, there is no signal at all impinging on the transponder and, therefore, no retransmitted signal to the base station. This limitation could be removed by mounting the tag in vertical position (as in Riley and Smith 2002) and consequently adopting antennas with vertical or circular polarization. An alternative solution, which requires no hardware modifications in terms of antennas, is instead the one depicted in Figure 6 on the right; with a “cross" dual tag configuration, no matter which is the relative position of the insect with respect to the radar, there is at least one visible transponder and hence a response from the target. Figure 7 reports on the right how this kind of transponder is mounted on the yellow‐legged hornet: the dual diode tag with two looped wires orthogonally glued together is tied to the insect with the help of a cotton strand. We redirect the reader to the results section for a detailed analysis of the pros and cons of all configurations.

#### The RX antenna

**Figure 8:**
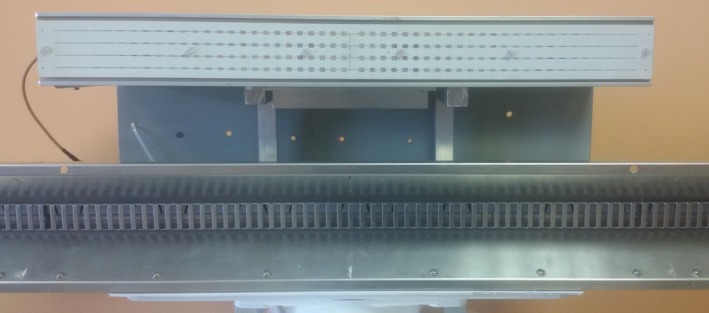
Receiving antenna (50 cm long) designed realized in microstrip on top of a portion of the transmitting slotted waveguide antenna.

As previously anticipated, the RX antenna, similarly to the TX launcher, works in horizontal polarization; however, while the FURUNO radar is equipped with a slotted waveguide, we preferred to design a microstrip cascade patch array at 18.82 GHz. Figure 8 shows the RX antenna in its working position, that is, mounted on top of the TX antenna.

**Figure 9:**
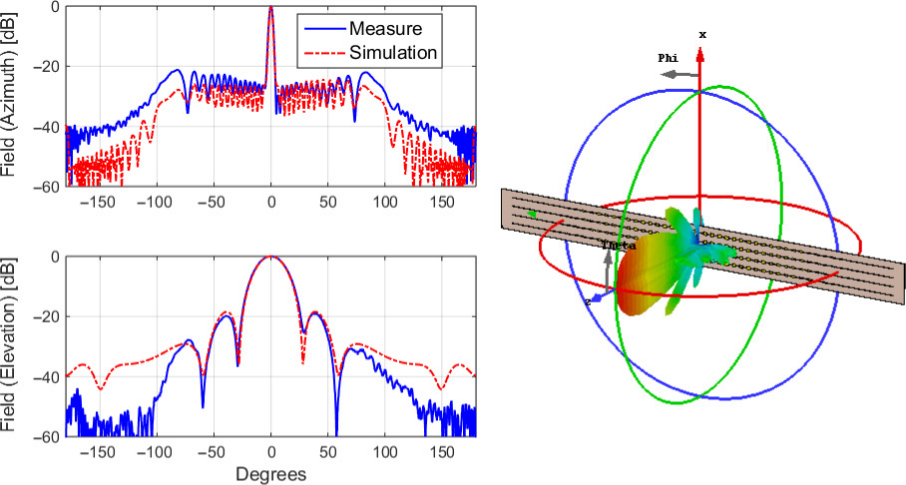
Comparison of the radiated fields in azimuth (top left) and elevation (bottom left) between the CST‐MWS simulated design (dashed red curve) and the measured data (plain blue curve), and 3D rendering of the simulated performances (right).

This antenna is realized on a 20 mils ROGER RO4350 dielectric layer, and it is basically a 4 × 50 patch array; the radiating elements are fed in cascade and produce a very narrow HPBW in the azimuthal plane, namely 2.4${}^{\circ }$, and a broader HPBW in elevation, namely 22.8${}^{\circ }$, with a gain of 27.4 dBi along the direction of maximum radiation. Figure 9 reports the comparison of the radiated fields between the CST‐MWS (http://www.cst.com) simulated design and the manufactured launcher for both planes, together with a 3D simulated rendering.

#### The RX hardware

The first module of the receiving chain is the LNB block, a NORSAT amplifier, and down‐converter (9000HX‐O3B‐BN KA‐BAND DUO LNB; http://www.norsat.com/wp-content/uploads/9000hx-o3b_lnb.pdf) working into the 18.372–19.300 GHz band; it is equipped with a local oscillator (LO) at 17.4 GHz and shows a minimum gain of 55 dB with a maximum noise figure of 1.5 dB. The LNB first amplifies the input signal and then down converts it to 1420 MHz.

Right after the LNB, the signal is filtered with a home‐designed coupled‐line bandpass filter centered at 1420 MHz, with a bandwidth of about 200 MHz at −3 dB. The filter reduces the noise going to the next stage, that is, to the detector.

The detector is an evaluation board from Analog Devices equipped with an AD8318 (http://www.analog.com/media/en/technical-documentation/evaluation-documentation/AD8318.pdf) logarithmic detector with 70 dB of input range, able to detect pulses as low as −65 dBm of power. It rectifies received pulses modulated from 1 MHz to 8 GHz and shows a 10 ns fast pulse rise and a 12 ns fall time.

To conclude the hardware analysis, the resulting signal is then processed by the DSP board.

It is first sampled with a 16‐bit analog‐to‐digital converter (ADC) working at 100 MHz, with a 1.2 V (peak to peak) range. The board is then equipped with an FPGA, which buffers the ADC sampled data and streams them to the central processing unit (CPU), a C6455 DSP fixed‐point at 1 GHz from Texas Instruments (http://www.ti.com/lit/ds/symlink/tms320c6455.pdf), with 1 GB of DDR3 RAM. The CPU performs the coded algorithm and sends the computed results to an external laptop via a Bluetooth connection. An Hall effect magnetic sensor, connected to one of the CPU general‐purpose input/output (GPIO), is also used to get information about the radar azimuth orientation.

**Figure 10:**
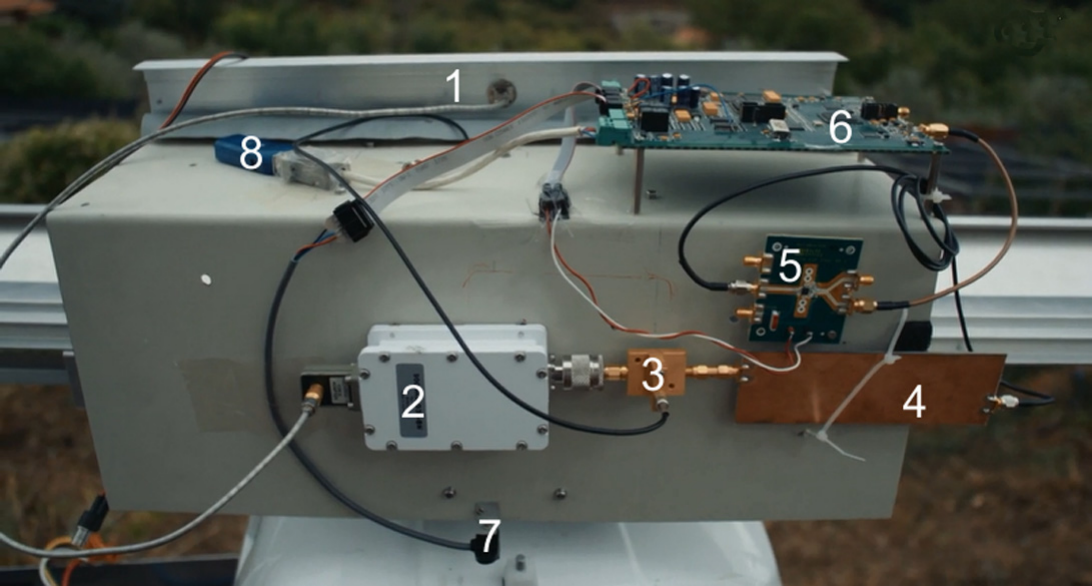
Summary of the hardware adopted for the receiving chain as it is mounted on the final system. (1) back of the RX antenna; (2) low‐noise down‐converter block; (3) bias tee, that is, feeder of the low‐noise block; (4) bandpass filter; (5) logarithmic detector; (6) digital signal processor; (7) Hall effect magnetic sensor; (8) Bluetooth transceiver.

Figure 10 shows a picture of the adopted RX hardware as it is installed on the harmonic radar system.

#### The RX software

The pulse leakage from the TX transmitter triggers the board to capture and elaborate real‐time data. To increase the processing gain, the DSP usually averages 16 subsequent pulse sequences and execute a correlation in range to reduce noise. A moving target identification (MTI, see Skolnik 1962 in chapter 3) algorithm was also developed to filter out leaked pulses and clutter echoes, which are quite strong especially from buildings, vehicles, and metallic objects in general. The received pulses greater than a fixed threshold value are reported over a polar plot centered at the radar location. The received signal is sampled at 100 MSPS with about 3600 pulse sequences captured per revolution, for an accuracy in range of 1.5 m (out of a raw radar resolution of 15 m) and in angle of 0.1${}^{\circ }$.

**Figure 11:**
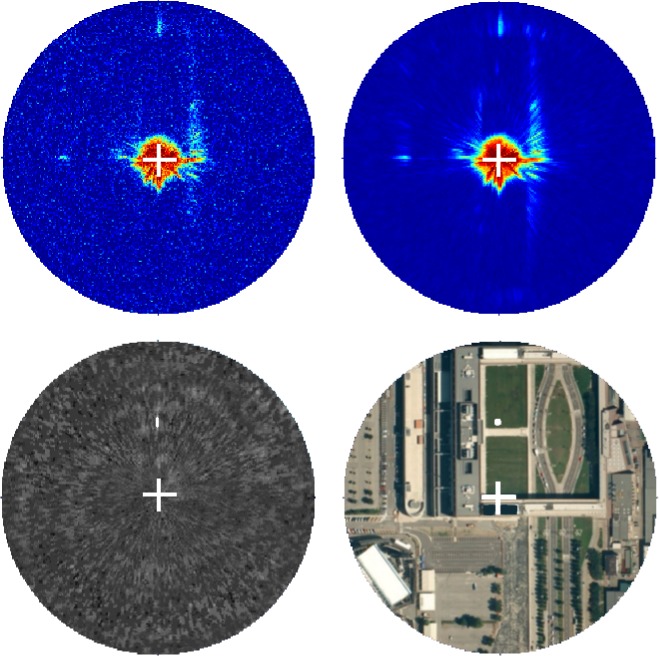
Received data at the different stages of the processing algorithm: raw signal (top left), averaged signal (top right), moving target identification (MTI) results (bottom left), and satellite map (bottom right). The boundaries of the polar plots correspond to 180 m and the target is located approximately 100 m in front of the radar system, which is positioned at the center of the plots and marked with a cross). The average process permits to discriminate relevant signals from background noise, while the MTI algorithm removes all steady obstacles.

A typical acquisition is documented in Figure 11, where the boundaries of the polar plots correspond to 180 m and the target is located approximately 100 m in front of the radar system (positioned at the center of the plots and marked with a cross). The top left plot shows the raw data, that is, all the detected signals by the radar. The top right graph reports the received data after the averaging process previously mentioned: At this stage, only the obstacles with the higher radar cross section remain (among which our target too). In this specific case, a metallic fence on the right and a building on the left are quite clearly visible in the plot, together with a number of obstruction right at the back of the system. The bottom left picture documents the effect of the MTI algorithm: Being all the aforementioned obstacles steady elements, the applied algorithm practically removes all of them with the exception of the target, which is obviously kept in movement. The final plot locates in white the radar and the target position on a satellite map (taken from http://www.bing.com/maps/).

## Results

Before proceeding with the description of the tracking set‐up and the obtained results, we would like to provide here the analysis of the expected performances. According to the radar equation, the received power $P_{\mathrm{RX}}$ can be expressed as:

(2) $$P_{\mathrm{RX}}=P_{\mathrm{TX}}G_{\mathrm{TX}}G_{\mathrm{RX}}\sigma_{h}\frac{1}{4\pi }{\left(\frac{\lambda_{h}}{4\pi R^{2}}\right)}^{2}$$

where $P_{\mathrm{TX}}$ is the transmitted power, $G_{\mathrm{TX}}$ and $G_{\mathrm{RX}}$ are the gain of the transmitting and receiving antennas, respectively, $\sigma_{h}$ is the measured harmonic cross section of the transponder, *R* is the distance from antennas to the transponder, and $\lambda_{h}$ is the wavelength at harmonic frequency.

The minimum received power required for the detection is:

(3) $$P_{\min}=kTBN_{f}V_{f}$$

where *k* is the Boltzmann's constant, *T* is the absolute temperature, *B* is the receiver bandwidth in Hz, $N_{f}$ is the noise figure of the LNB, and $V_{f}$ is a visibility factor to detect signal from noise with an acceptable false alarm probability. The maximum detection range of the system can be finally computed as:

(4) $$R_{\max}={\left(\frac{\lambda_{h}}{4\pi }\right)}^{0.5}{\left(\frac{P_{\mathrm{TX}}G_{\mathrm{TX}}G_{\mathrm{RX}}\sigma_{h}}{4\pi P_{\min}}\right)}^{0.25}$$

Using the values listed in Table 2, one can estimate a maximum detection range of about 120 m; this is a conservative value, as the conversion gain of the diode has been estimated at a lower power density than in the operative conditions.

**Table 2:** Performance of the different components of the developed harmonic radar system, required to compute the maximum expected detection range

$P_{\mathrm{TX}}$	25 kW (74.0 dBm)
$G_{\mathrm{TX}}$	707.9458 (28.5 dBi)
$G_{\mathrm{RX}}$	549.5409 (27.4 dBi)
$\sigma_{h}$	$2\;\times \;10^{-6}$ mm${}^{2}$
*B*	200 MHz
$N_{f}$	1.4125 (1.5 dB)
*T*	290 K
*k*	$1.38\;\times \;10^{-23}$/WHz/K
$V_{f}$	10 (10 dB)

On‐field tests were performed nearby Dolceacqua, in the inland of the Ligurian coast, few kilometers from the France border. A small cluster of honeybee hives was attacked by a conspicuous group of yellow‐legged hornets this summer, allowing us to capture and tag many specimens during the testing phase of the radar. The area under analysis was about 300 m × 300 m, presented a not negligible slope of approximately 10% and had a discrete amount of tall trees and obstacles (poles, fences, houses, etc.)

The testing procedure was structured as follows:

1. Preliminary check of the system without any target in order to find the best settings for the environment under analysis.
2. Detection of a tag mounted on a drone in order to find the maximum range of the radar in that specific context and, in case, some occluded areas; for this task, a single “loop" transponder was adopted, but the drone was operated leaving the tag in the most favorable position during the entire flight.
3. Detection of multiple targets. This task comprised the capture of the hornet in front of the hives, the fastening of the transponder to the anaesthetized insect (usually by a short exposure to cold temperatures), the release of the hornet and the tracking of its flight.

**Figure 12:**
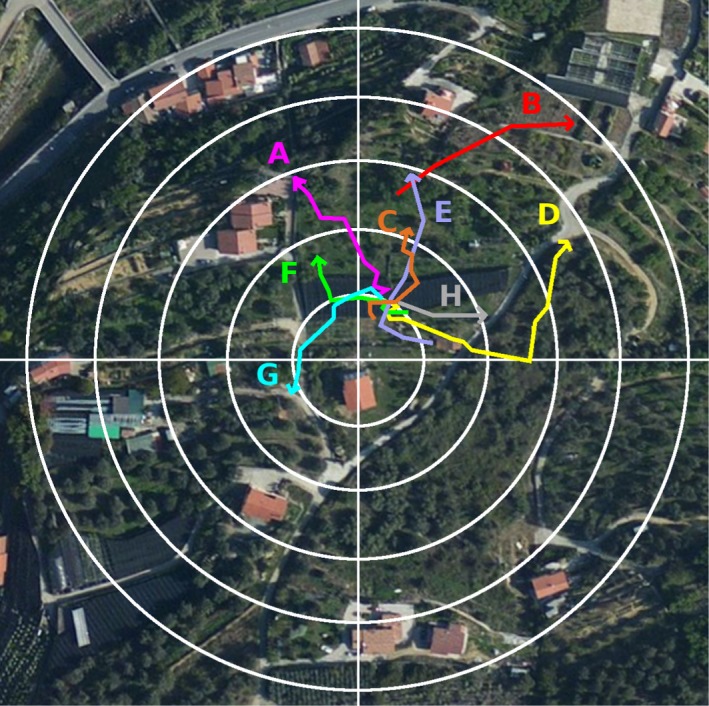
Examples of tracked insect flights during the on‐field campaign. The arrow identifies the last detected point of the insect flight, while the distance between two consecutive white circles corresponds to 25 m. The accuracy in range is 1.5 m out of a raw radar resolution of 15 m.

Figure 12 reports a set of not‐simultaneous seven traces; the distance between two consecutive white circles corresponds to 25 m. The reader can immediately notice that the initial maximum detection was slightly <125 m (A) and the flight trajectory seemed to indicate a certain preference for north (A,C,E,F) and northeast (B,D) directions. On average, as expected we found a better signal reflection when the “cross" tag was mounted (A,B,D); however, we also noticed that in few cases (for instance C) the hornet got rid of the tag quite immediately, simply cutting the cotton strand as soon as it temporarily landed. In case of “loop" tag glued on the back of the insect, we could also observe few wasps coming back to the honeybee hives two days after the release, this indicating that the position of the tag is of no obstacle to their flight and, in general, to their “normal" life (for instance they can enter their nest). Finally, the radar was also able to detect tagged insect flying at about 15 m from the soil, that is, above some of the tall trees present on the field.

## Discussion

The aim of this work is to restrain the diffusion of the yellow‐legged Asian hornet in a hilly and woody land such as the Western Liguria; that being so, we designed an harmonic radar system able to track the flight of the *V. velutina* specimens, once equipped with proper transponder, in order to locate their nests in that kind of complex environment.

Harmonic radars for insect tracking have been used in many applications so far. Portable systems such as *Recco* (for instance the ones described in Mascanzoni and Wallin 1986; Brazee et al. 2005; Hall and Hadfield 2009) provide rather inaccurate range data, and they are limited in distance (few meters) and they can monitor only slow moving targets. They are mainly adopted as supporting tools for capture–mark–recapture operations in limited areas and, hence, they are not useful for our purpose. Few works on low‐power and high‐distance radars have been recently released, for instance the one described in Tsai et al. (2013), where advanced transmission techniques such as the binary phase‐shift keying (BPSK) modulation allowed to increase the range distance and reduce the input power with respect to the previously mentioned references. Nevertheless, to the best of our knowledge, the only paper successfully documenting a detection above 500 m is Riley et al. (1996), described in detail in Riley and Smith (2002), and adopted as reference also by Osborne et al. (1999).

For this reason, starting from Riley and Smith (2002), we upgraded that proposal to meet our requirements. We first increased the vertical beam of the antennas in order to be able to operate on hilly and rich in obstacles areas. We then adopted a digital elaboration system of the received signal, namely through DSP, to increase the detection probability, to memorize the traces in a statistical way, and to have an accurate precision of detection in range and angle. As pointed out by Boiteau et al. (2011), the absence of a clear line of sight, as it may happen in a place full of vegetation, makes the detection far more difficult; however, by means of a continuous and prolonged detection of the single traces, it is possible to reconstruct preferential directions of flight of the tagged insects.

By taking into account the gain of each component, we estimated a theoretical maximum range of about 120 m. Our system proved to work as expected during on‐field tests, despite the complexity of the location in terms of obstacles and slope, reaching a maximum distance of detection of approximately 125 m. This value is slightly higher, basically for two reasons. First, the implemented digital filtering techniques permitted to increase the detection capabilities of the radar, leaving unchanged the probability of false alarms. Second, the transponder at 125 m received a higher power density with respect to the one measured in laboratory and showed a higher conversion efficiency than the estimated one.

One element which clearly needs more study is the positioning of the transponder on the hornet. This operation not only has to be quick and simple to be accomplished, but it also has to maximize the detection probability; eventually, the tag should not cause any interference to the insect everyday activities. This last issue is even more important if one considers the possibility to extend the tracking throughout few days or move the harmonic radar along the preferential trajectory of the insects in order to locate their nests. That being so, a foreseen improvement of the system will be the adoption of circularly polarized antennas, which will definitely increase detections and will allow, for instance, to investigate the behavior of the tag as mounted in Riley and Smith (2002).

Similarly, also the maximum detection range can be improved with the help of a narrower band receiver and with the reduction of the noise floor. For instance, with a bandwidth of 20 MHz, as the one described in Riley and Smith (2002), our system will have a theoretical maximum distance of about 216 m.

Finally, to simplify the radar handling, the system will be installed on a movable telescopic tower which will allow to run it from an elevated position (about 3 m from the soil); this new possibility will likely permit to get rid of some of the short trees which sometimes determine the abrupt interruption of the insect trace. Furthermore, once the system will be correctly positioned on the tower, it will be also easier to move it according to the preferential trajectories.

Once the possibility to follow the flight of the yellow‐legged hornet and to locate its nests by means of an harmonic radar system will be fully mastered, it will be possible to develop more advanced systems which may include more elaborated transmission schemes, as the BPSK outlined in Tsai et al. (2013), in order to obtain more compact instruments. At that point, in the presence of yellow‐legged hornet specimens, the restrain procedure will be as follows: A trained team will install the next‐generation radar system nearby a honeybee hive under attack, it will tag a number of insect and track their flight to get to their nest and finally destroy it.
